# HMOX1 Attenuates the Sensitivity of Hepatocellular Carcinoma Cells to Sorafenib via Modulating the Expression of ABC Transporters

**DOI:** 10.1155/2022/9451557

**Published:** 2022-06-27

**Authors:** Xian Zhu, Yinfang Zhang, Yafei Wu, Wenjing Diao, Guilong Deng, Qin Li, Chuanxing Wu

**Affiliations:** ^1^Department of Critical Care Unit, Shanghai General Hospital, Shanghai Jiaotong University, Shanghai, China; ^2^Department of Nursing, Shanghai General Hospital, Shanghai Jiaotong University, Shanghai, China; ^3^Department of Clinical Pharmacy, Shanghai General Hospital, Shanghai Jiao Tong University School of Medicine, Shanghai, China; ^4^Department of General Surgery, Shanghai General Hospital, Shanghai Jiaotong University, Shanghai, China

## Abstract

Hepatocellular carcinoma (HCC) represents a common malignancy, and mechanisms of acquired sorafenib resistance during the treatment of HCC patients remain elusive. The present study performed integrated bioinformatics analysis and explored the potential action of heme oxygenase 1 (HMOX1) in sorafenib-resistant HCC cells. Differentially expressed genes (DEGs) of the sorafenib-resistant group as compared to the sorafenib-sensitive group from GSE140202 and GSE143233 were extracted. Fifty common DEGs between GSE140202 and GSE143233 were extracted. Ten hub genes were identified from the protein-protein interaction network based on common DEGs. Experimental results revealed the upregulation of HMOX1 in sorafenib-resistant HCC cells. HMOX1 silence promoted the sensitivity to sorafenib in sorafenib-resistant HCC cells; overexpression of HMOX1 attenuated the sensitivity. In addition, HMOX1 silence downregulated the mRNA expression of ABC transporters in sorafenib-resistant HCC cells, while HMOX1 overexpression upregulated mRNA expression of ABC transporter expression in HCC cells. Further analysis also revealed that high expression of HMOX1 was associated with shorter OS and DSS in HCC patients. In conclusion, our analysis identified ten hub genes associated with sorafenib resistance in HCC. Further validation studies demonstrated that HMOX1 promoted sorafenib resistance of HCC cells via modulating ABC transporter expression.

## 1. Introduction

Hepatocellular carcinoma (HCC) represents a common tumor malignancy, and HCC incidence is expected to increase annually [1, 2]. Among all the cancer types, HCC is one of the most frequently diagnosed malignancies around the world [1, 2]. The HCC patients' overall survival (OS) remains low due to insufficient early diagnosis and the recurrence and metastasis of advanced HCC [3–5]. In terms of early diagnosis of HCC, imaging has limitations in diagnostic accuracy and sensitivity, while common serum markers show poor diagnostic performance [6]. With the advent of high-throughput sequencing technology, genetic biomarkers such as cell-free DNA (cfDNA), epigenetic changes, and circulating RNA (microRNAs (miRNAs), long noncoding RNAs (lncRNA), and circular RNAs (circRNA)) from peripheral blood have become the focus of the early diagnosis of HCC [7]. However, there are still limitations in the early diagnosis of HCC. The main limitation of most studies using nucleic acid molecules as biomarkers of HCC is the limited size of the cohort, and the heterogeneity of HCC should be also considered. Sorafenib was first approved by the FDA for HCC therapy at advanced stages. Sorafenib treatment could significantly improve the OS of patients with HCC by 2-3 months. Unfortunately, many patients with HCC had a poor response to sorafenib or exhibited sorafenib resistance after prolonged use of sorafenib [8–10]. The molecular mechanisms underlying the acquired sorafenib resistance are complex which may involve epithelial-mesenchymal transition, tumor microenvironment, autophagy, and cancer stem cells [10–12]. Besides, studies also proposed that acquired resistance to sorafenib may be associated with the dysregulated signaling pathways including JAK/STAT, PI3K/Akt, and TNF*α*/NF-*κ*B [10–12]. Thus, it is urgent to fully decipher mechanisms associated with sorafenib resistance, which may provide a better therapeutic strategy for treating advanced HCC.

With the rapid development and progress in the high-throughput technologies, the investigation of tumor biology has been focusing on the genomic scale. RNA sequencing has been widely used in identifying novel targets in cancer pathophysiology, including HCC. For instance, Weng et al. performed the RNA-sequencing analysis in HCC and sorafenib-resistant HCC tissues and identified novel cricFOXM1 as crucial modulator of sorafenib resistance in HCC cells [13]. Wu et al. also carried out global transcriptomic sequencing in sorafenib-resistant HCC cells and emphasized the importance of circRNAs in mediating sorafenib resistance [14]. Wu et al. performed the RNA-sequencing analysis and revealed that mitophagy promoted sorafenib resistance through hypoxia-inducible ATAD3A dependent axis [15]. RNA-sequencing studies also proposed that epigenetically activated ADAMTSL5 is a key player in HCC drug resistance [16]. In addition, the integrated bioinformatics analysis of publicly available microarray datasets also provided another strategy for scientists to discover novel mediators associated with the acquired sorafenib resistance in HCC. Liu et al. analyzed dataset GSE109211 and proposed that HCC sorafenib resistance was correlated with identified hub genes and pathways [17]. Jiang et al. analyzed GSE62813, GSE73571, GSE151412, and GSE140202 and found other key mediators in HCC sorafenib resistance [18].

This study employed a strategy to integrate two datasets (GSE140202 and GSE14322) from the GEO database and extracted the DEGs between sorafenib-sensitive and sorafenib-resistant groups. The extracted DEGs were processed for PPI network construction and functional analysis. Furthermore, HMOX1, one of the hub genes, was further validated in sorafenib-resistant HCC cells, and the potential action of HMOX1 in sorafenib-resistant HCC cells was also preliminarily explored.

## 2. Materials and Methods

### 2.1. Extraction of GEO RNA-Sequencing Datasets

RNA-sequencing datasets were retrieved from the public GEO database. In the analysis, we searched the RNA-sequencing datasets profiling the mRNA expression between sorafenib-sensitive and sorafenib-resistant HCC cells, and samples < 3 in each group were excluded in the analysis. After screening the processed GEO RNA-sequencing datasets using GREIN-iLINCS tool [19], two RNA-sequencing datasets, including GSE140202 and GSE143233, were finally included in our analysis. GPL20795 HiSeq X Ten and GPL16791 Illumina HiSeq 2500 platforms were used in GSE140202 and GSE143233, respectively. In GSE140202, 6 sorafenib-sensitive HCC cells (*n* = 6) and sorafenib-resistant HCC cells (*n* = 6) were included for analysis. In GSE143233, three samples of HCC tissues and three samples of sorafenib-resistant HCC tissues were included for analysis.

### 2.2. Extraction of DEGs in the Collected RNA-Sequencing Datasets

The extraction of DEGs in GSE140202 and GSE143233 was performed using the GREIN-iLINCS tool [19]. The DEGs between sorafenib-sensitive and sorafenib-resistant HCC cells were extracted in GSE140202; the DEGs between HCC and sorafenib-resistant HCC tissues were extracted for GSE143233. The significant DEGs were extracted using the following criteria: log|FC| > 1.2 and *P* values < 0.05. The heatmaps of DEGs in GSE140202 and GSE143233 were plotted by using the GREIN-iLINCS tool, and the top 200 DEGs were illustrated in the heatmaps. The R software plotted the volcano plots of the DEGs in GSE140202 and GSE143233 with ggplot function. The Venn diagrams showing the common DEGs between GSE140202 and GSE143233 were also generated using R software.

### 2.3. Functional Analysis of DEGs between GSE140202 and GSE143233

The GO functional enrichment and KEGG pathway enrichment analyses of common DEGs between GSE140202 and GSE143233 were carried out using EnrichR, an interactive and collaborative HTML5 gene list enrichment analysis tool.

### 2.4. PPI Network Construction Based on Common DEGs Extracted from GSE140202 and GSE143233

PPI network of the common DEGs between GSE140202 and GSE143233 was generated by STRING [20]. The PPI network was visualized by using Cytoscape software. To further extract the key submodules in the PPI network, the cytoHubba application from Cytoscape software was applied to visualize submodules derived from the PPI network.

### 2.5. Cell Lines and Cell Culture

The Huh7 and HepG2 cells were obtained from ATCC (Manassas, USA). These cell lines were kept in Dulbecco's modified Eagle's medium (DMEM; Sigma) supplemented with 10% fetal bovine serum (FBS: Gibco) at 37°C in a humidified incubator containing 5% CO_2_. The generation of sorafenib-resistant HCC cells was performed by treating cells with 0.5 *μ*M sorafenib (Sigma) followed by escalating the dose to 10 *μ*M. Sorafenib-resistant Huh7 (Huh7-SR) and HepG2 (HepG2-SR) cells were maintained in DMEM containing sorafenib (10 *μ*M).

### 2.6. siRNAs and Plasmids

The siRNA that targets HMOX1 was obtained from RiboBio (Guangzhou, China). The scrambled sequence of HMOX1 siRNA was selected as the corresponding negative control (NC). The sequences for the corresponding siRNAs are shown in Supplementary Table S1. For HMOX1-overexpressing vectors, HMOX1 was ligated into the pcDNA3.1 vector to generate the HMOX1-expressing vector (RiboBio), and the empty pcDNA3.1 vector was used as NC.

### 2.7. Cell Transfections

Cells (Huh7, Huh7-SR, HepG2, and HepG2-SR cells) were seeded at 5 × 10^5^ cells per well in 6-well plates in DMEM containing 10% FBS overnight. Transfection experiments were performed with OPTI-MEM serum-free medium and Lipofectamine 2000 reagent with respective siRNA (scrambled siRNA or HMOX1 siRNA) or vectors (pcDNA3.1 or pcDNA3.1-HMOX1). At 24 h post-siRNA or vector transfection, these cells were harvested for other experimental procedures.

### 2.8. Cell Viability Assay

The cell viability of respective HCC cells was measured by the Cell Counting Kit-8 (CCK-8) assay (Beyotime, Beijing, China). Briefly, respective HCC cells after siRNA/vector transfections and/or treatment with incremental concentrations of sorafenib were incubated with 10% CCK-8 in DMEM for 4 h. The cell viability was measured by calculating absorbance at 450 nm with a plate reader.

### 2.9. Quantitative Real-Time PCR

Total RNA was isolated using Trizol reagent (Qiagen, Hilden, Germany). A NanoDrop spectrophotometer was applied to measure RNA concentration (Thermo Fisher Scientific). Five *μ*g RNA was transcribed into cDNA with a reverse transcription kit (Applied Biosystems). StepOnePlus Real-Time PCR System was used to perform real-time PCR (Applied Biosystems). Thermal conditions of amplification on cycles were as follows: 95°C for 2 min, 40 cycles at 95°C for 30 s, 56°C for 30 s, and 72°C for 1 min, then 72°C for 5 min. GAPDH was used as an internal control. Relative expression of the corresponding mRNA was calculated using the 2^-*ΔΔ*Ct^ method. The primer sequences are shown in Supplementary Table 2.

### 2.10. Survival Analysis of Patients with HCC

The impact of HMOX1 on OS and disease-specific survival (DSS) of HCC patients was evaluated by the Kaplan Meier plotter [21]. Log-rank *P* < 0.05 was statistically significant.

### 2.11. Statistical Analysis

The statistical analysis was carried out by GraphPad Prism 5 software (GraphPad Software). All the data were displayed as mean ± standard deviation. Unpaired Student's *t*-test determined significant differences between treatment groups. *P* < 0.05 was statistically significant.

## 3. Results

### 3.1. Analysis of DEGs between Sorafenib-Sensitive and Sorafenib-Resistant Groups in GSE140202 and GSE143233 Datasets

**Figure 1:**
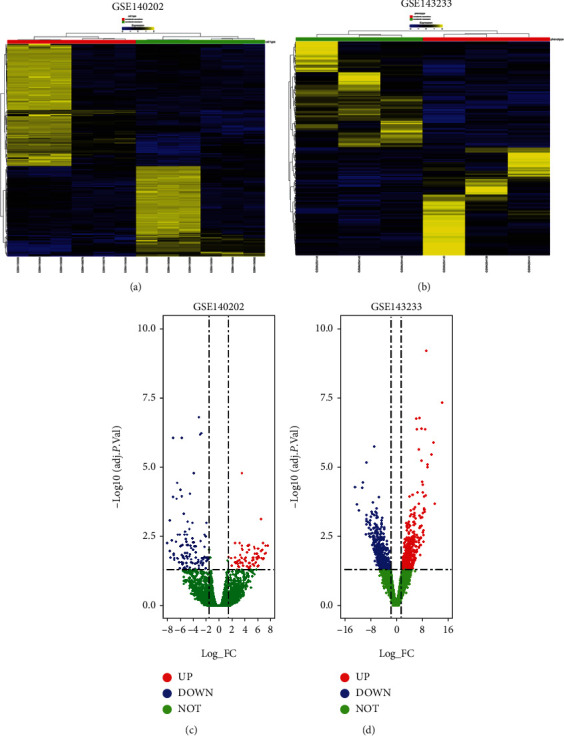
Heatmap and volcano plots of DEGs in GSE140202 and GSE143233. Heatmaps of the DEGs in (a) GSE140202 and (b) GSE143233 are shown. The volcano plots of DEGs in (a) GSE140202 and (b) GSE143233 are shown. UP: upregulated genes (red dots); DOWN: downregulated genes (blue dots); NOT: not significantly expressed genes (grey dots).

**Figure 2:**
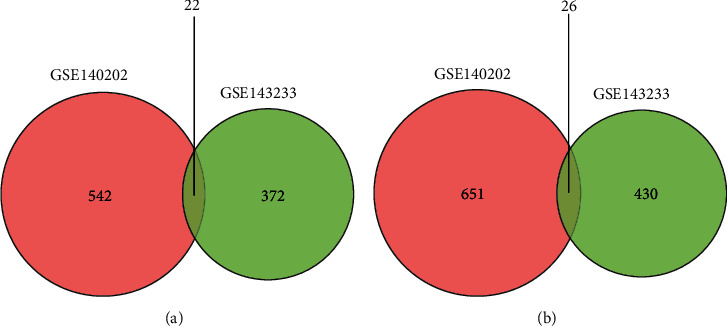
Venn diagram of common DEGs in GSE140202 and GSE143233. (a) A Venn diagram of common upregulated DEGs in GSE140202 and GSE143233 is shown. (b) A Venn diagram of common downregulated DEGs in GSE140202 and GSE143233 is shown.

The DEGs between two groups in GSE140202 and GSE143233 were plotted as heatmaps (Figures 1(a) and 1(b)) and volcano plots (Figures 1(c) and 1(d)). In GSE140202, 542 upregulated and 651 downregulated DEGs were extracted between two groups in GSE140202; in GSE143233, 372 upregulated DEGs and 430 downregulated DEGs were extracted between two groups in GSE143233. The Venn diagram showed that 22 upregulated common DEGs were found between GSE140202 and GSE143233 (Figure 2(a)); 26 downregulated common DEGs were found between GSE140202 and GSE143233 (Figure 2(b)).

### 3.2. Functional Analysis of Common DEGs

**Figure 3:**
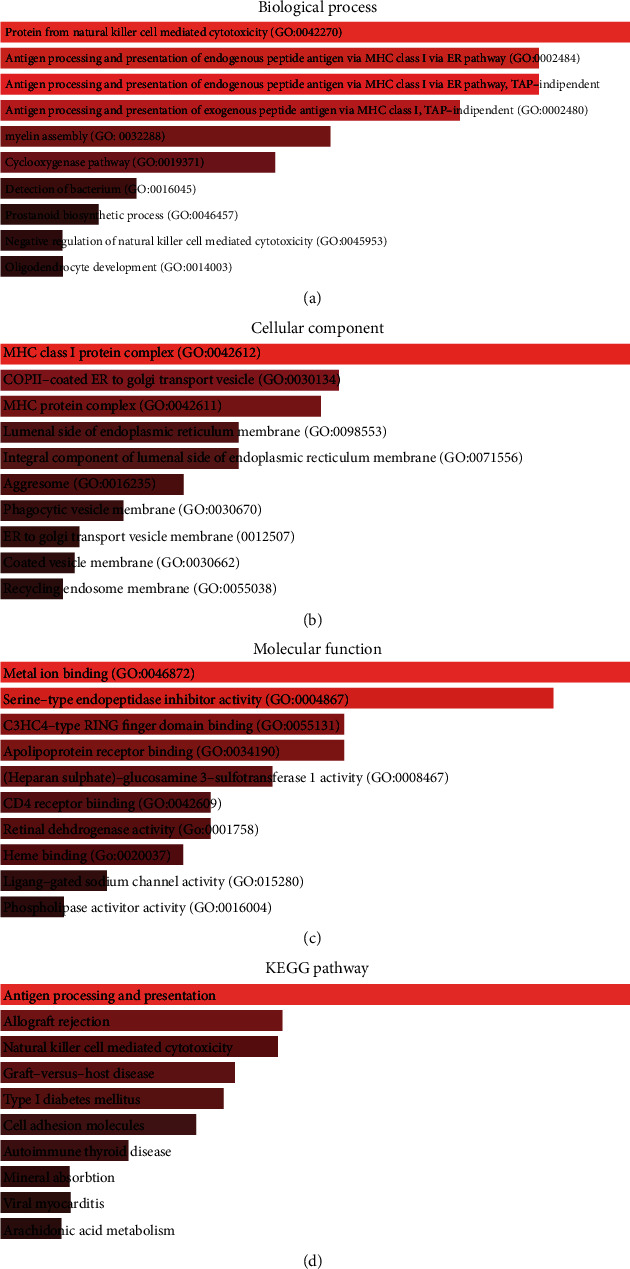
Functional analysis of common DEGs in GSE140202 and GSE143233. (a) The GO_biological process enrichment analysis of common DEGs in GSE140202 and GSE143233. (b) The GO_cellular component enrichment analysis of common DEGs in GSE140202 and GSE143233. (c) The GO_molecular function enrichment analysis of common DEGs in GSE140202 and GSE143233. (d) The KEGG pathway enrichment analysis of common DEGs in GSE140202 and GSE143233.

Common DEGs in GSE140202 and GSE143233 were further processed for functional enrichment analysis. In the GO_biological process, common DEGs were enriched in GO terms such as “protection from natural killer cell mediated cytotoxicity”; “antigen processing and presentation of endogenous peptide antigen via MHC class I via ER pathway”; “antigen processing and presentation of endogenous peptide antigen via MHC class I via ER pathway, TAP-independent”; “antigen processing and presentation of exogenous peptide antigen via MHC class I, TAP-independent”; and “myelin assembly” (Figure 3(a)). In the GO_cellular component, common DEGs were enriched in GO terms such as “MHC class I protein complex,” “COPII-coated ER to Golgi transport vesicle,” “MHC protein complex,” and “lumenal side of endoplasmic reticulum membrane” (Figure 3(b)). In GO_molecular function, common DEGs were significantly enriched in the GO terms such as “metal ion binding,” “serine-type endopeptidase inhibitor activity,” “C3HC4-type RING finger domain binding,” and “apolipoprotein receptor binding” (Figure 3(c)). In KEGG pathway analysis, common DEGs were enriched in “Antigen processing and presentation,” “Allograft rejection,” “Natural killer cell mediated cytotoxicity,” and “Graft-versus-host disease” pathways (Figure 3(d)).

### 3.3. PPI Network Analysis from Common DEGs in GSE140202 and GSE143233

**Figure 4:**
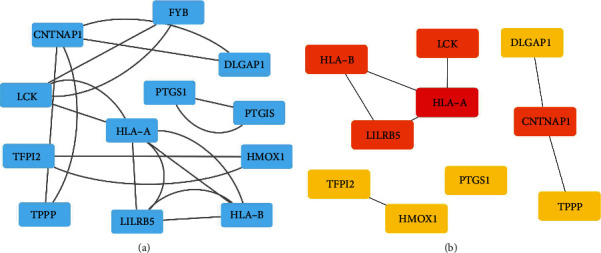
PPI network analysis of common DEGs in GSE140202 and GSE143233. (a) PPI network of DEGs. (b) Submodules of DEGs were extracted by cytoHubba of Cytoscape software.

PPI network was established based on 48 common DEGs. The 12 nodes and 18 edges were detected in constructed PPI network (Figure 4(a)). Furthermore, the cytoHubba analysis extracted four submodules. The most significant submodule contains four nodes and four edges (Figure 4(b)).

### 3.4. HMOX1 Exhibited an Upregulation in the Sorafenib-Resistant HCC Cells

**Figure 5:**
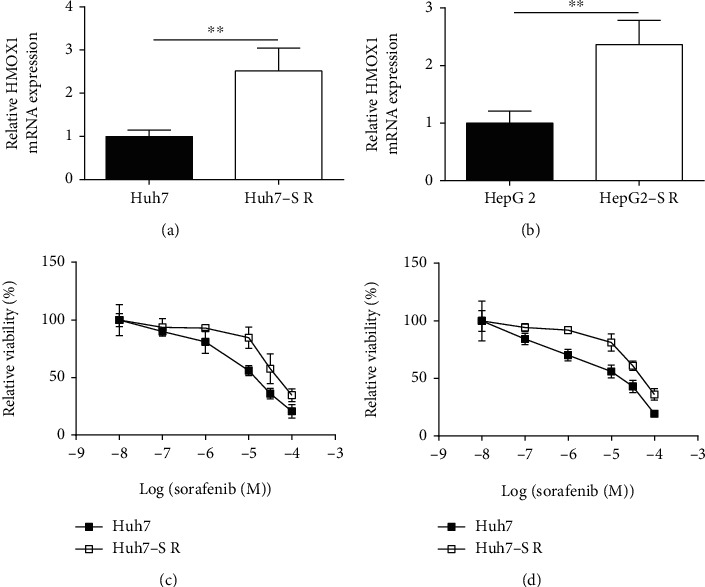
HMOX1 was upregulated in the sorafenib-resistant HCC cells. (a) The relative mRNA expression of HMOX1 in Huh7 and Hun7-SR cells. (b) The relative mRNA expression of HMOX1 in HepG2 and HepG2-SR cells. (c) MTT assay measured the relative viability of Huh7 and Huh7-SR cells. (d) MTT assay measured the relative viability of HepG2 and HepG2-SR cells. *N* = 3. ^∗∗^*P* < 0.01.

Based on the literature analysis, we proposed that HMOX1 may be a novel gene associated with sorafenib's sensitivity to the HCC cells. Thus, we compared HMOX1 mRNA expression in the normal HCC cell lines (Huh7 and HepG2) and the sorafenib-resistant HCC cell lines (Huh7-SR and HepG2-SR). HMOX1 was significantly upregulated in the sorafenib-resistant HCC cell lines (Huh7-SR and HepG2-SR) compared to their corresponding parental HCC cells (Figures 5(a) and 5(b)). Furthermore, the MTT assay showed that the suppressive actions of sorafenib on the cell viability were significantly attenuated in Huh7-SR and HepG2-SR cells compared to the corresponding parental cells (Figures 5(c) and 5(d)).

### 3.5. Actions of HMOX1 on Sorafenib Sensitivity of HCC Cells

**Figure 6:**
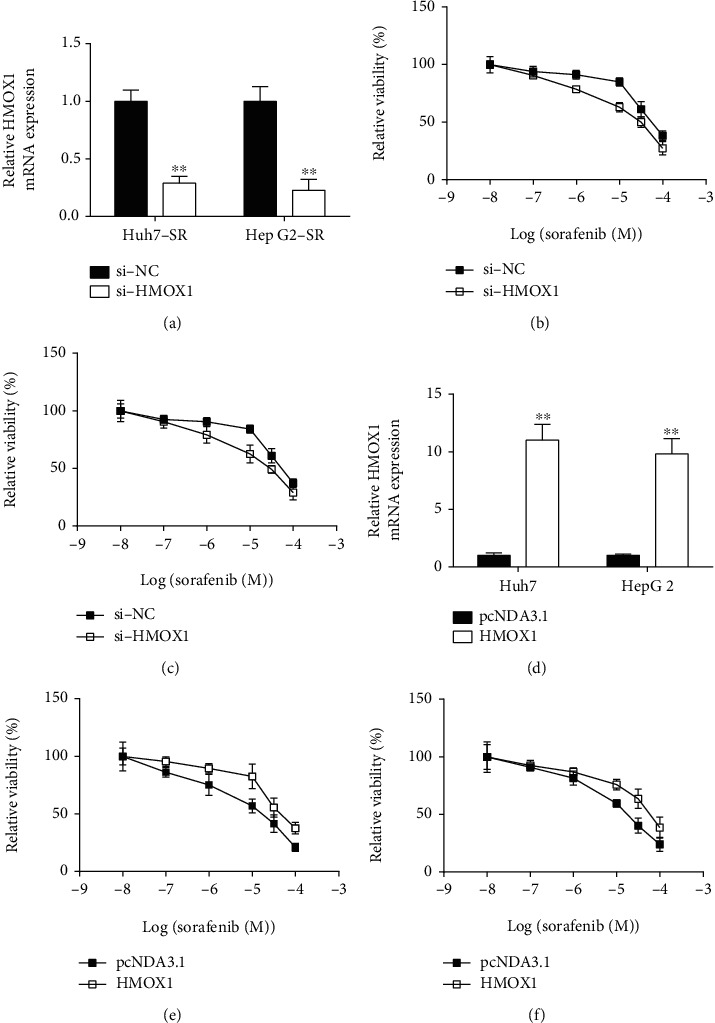
Effects of HMOX1 on the sensitivity of HCC cells to sorafenib. (a) The relative mRNA expression of HMOX1 in Huh7-SR and HepG2-SR cells after scrambled siRNA (si-NC) or HMOX1 siRNA (si-HMOX1) transfection. (b) The relative viability of scrambled siRNA (si-NC) or HMOX1 siRNA- (si-HMOX1-) transfected Huh7-SR cells after being treated by sorafenib was determined by MTT assay. (c) The relative viability of scrambled siRNA (si-NC) or HMOX1 siRNA- (si-HMOX1-) transfected HepG2-SR cells after being treated by sorafenib was determined by MTT assay. (d) The relative mRNA expression of HMOX1 in Huh7-SR and HepG2-SR cells after pcDNA3.1 or pcDNA3.1-HMOX1 transfection was determined by qRT-PCR. (e) The relative viability of pcDNA3.1 or pcDNA3.1-HMOX1 transfection-transfected Huh7-SR cells after being treated by sorafenib was determined by MTT assay. (f) The relative viability of scrambled pcDNA3.1 or pcDNA3.1-HMOX1 transfection-transfected HepG2-SR cells treated by sorafenib was measured by MTT assay. *N* = 3. ^∗∗^*P* < 0.01.

HMOX1 siRNA transfection significantly downregulated HMOX1 expression in Huh7-SR and HepG2-SR cells (Figure 6(a)). The MTT assay showed that silencing HMOX1 markedly promoted the sensitivity of Huh7-SR (Figure 6(b)) and HepG2-SR (Figure 6(c)) cells to sorafenib. Gain-of-function results revealed that pcDNA3.1-HMOX1 transfection induced an apparent increase in HMOX1 mRNA expression of Huh7 and HepG2 cells (Figure 6(d)). Functional assay results demonstrated that HMOX1 overexpression promoted sorafenib-resistance of Huh7 and HepG2 cells (Figures 6(e) and 6(f)).

**Figure 7:**
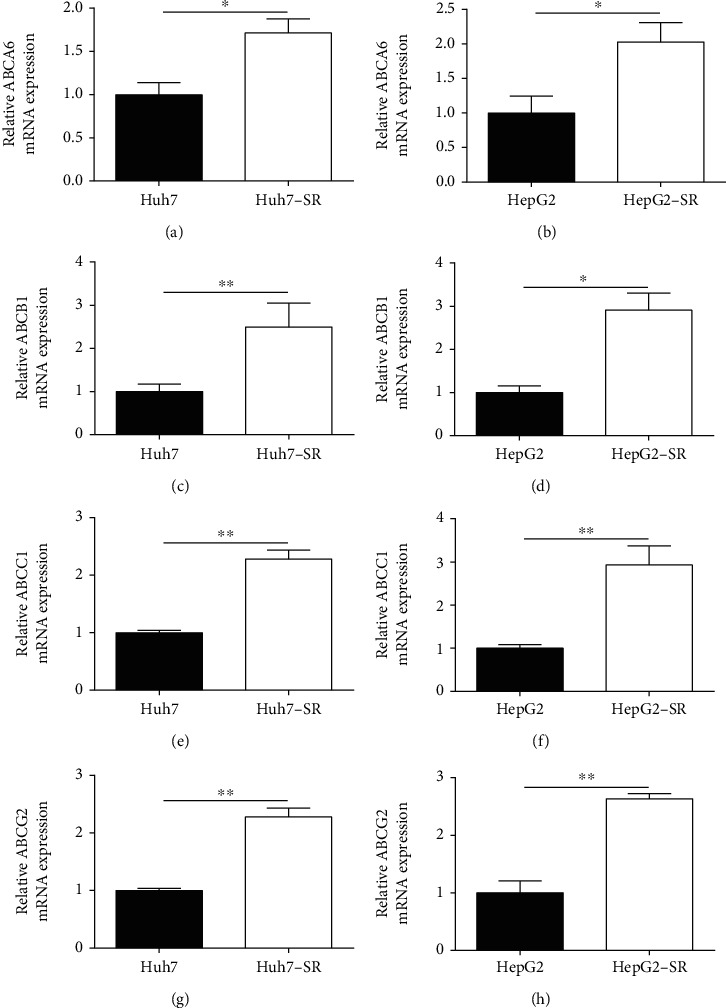
ABCA6, ABCB1, ABCC1, and ABCG2 expression was upregulated in the sorafenib-resistant HCC cell lines. (a, b) Relative mRNA expression of ABCA6 in Huh7, Huh7-SR, HepG2, or HepG2-SR cells. (c, d) Relative mRNA expression of ABCB1 in Huh7 and Hun7-SR cells. (e, f) Relative mRNA expression of ABCC1 in Huh7, Huh7-SR, HepG2, or HepG2-SR cells. (g, h) Relative mRNA expression of ABCG2 in Huh7, Huh7-SR, HepG2, or HepG2-SR. *N* = 3. ^∗^*P* < 0.05; ^∗∗^*P* < 0.01.

**Figure 8:**
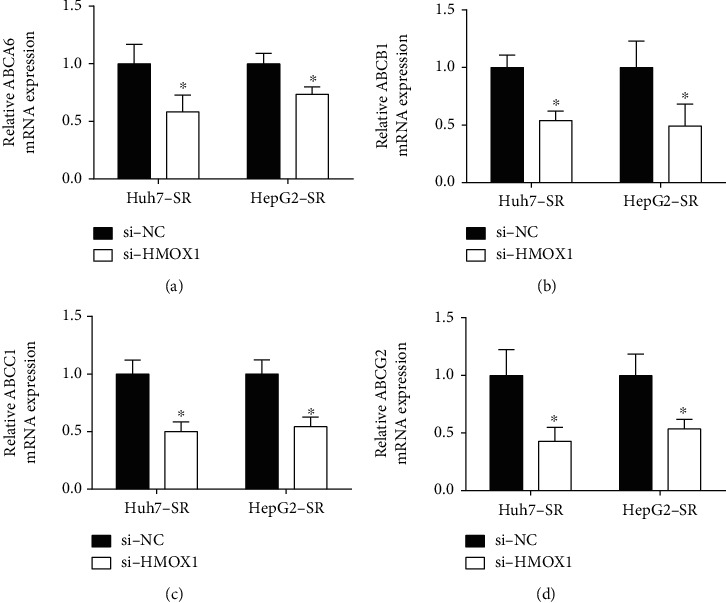
Silence of HMOX1 downregulated the expression of ABCA6, ABCB1, ABCC1, and ABCG2 in sorafenib-resistant cells. (a) Relative mRNA expression of ABCA6 in Huh7-SR and HepG2-SR cells after being transfected with si-NC or si-HMOX1. (b) Relative mRNA expression of ABCB1 in Huh7-SR and HepG2-SR cells after being transfected with si-NC or si-HMOX1. (c) Relative mRNA expression of ABCC1 in Huh7-SR and HepG2-SR cells after being transfected with si-NC or si-HMOX1. (d) Relative mRNA expression of ABCG2 in Huh7-SR and HepG2-SR cells after being transfected with si-NC or si-HMOX1. *N* = 3. ^∗^*P* < 0.05.

**Figure 9:**
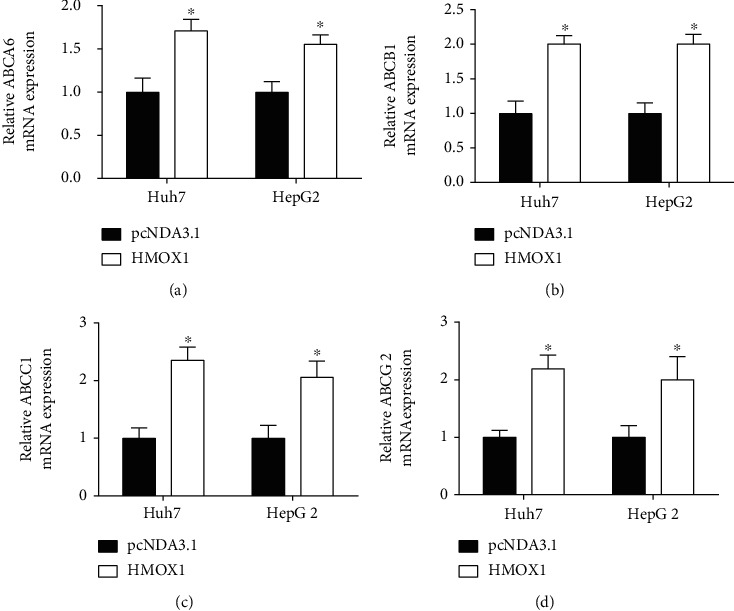
Overexpression of HMOX1 upregulated the expression of ABCA6, ABCB1, ABCC1, and ABCG2 in sorafenib-resistant cells. (a) Relative mRNA expression of ABCA6 in Huh7-SR and HepG2-SR cells after being transfected with pcDNA3.1 or pcDNA3.1-HMOX1. (b) Relative mRNA expression of ABCB1 in Huh7-SR and HepG2-SR cells after being transfected with pcDNA3.1 or pcDNA3.1-HMOX1. (c) Relative mRNA expression of ABCC1 in Huh7-SR and HepG2-SR cells after being transfected with pcDNA3.1 or pcDNA3.1-HMOX1. (d) Relative mRNA expression of ABCG2 in Huh7-SR and HepG2-SR cells after being transfected with pcDNA3.1 or pcDNA3.1-HMOX1. *N* = 3. ^∗^*P* < 0.05.

To further explore mechanisms associated with HMOX1-mediated sorafenib resistance of HCC cells, we examined actions of HMOX1 overexpression or silence on ABC transporter mRNA expression in HCC cells. The ABC transporter mRNA expression levels of ABCA6, ABCB1, ABCC1, and ABCG2 in these cells were upregulated compared to their corresponding parental HCC cells (Figure 7). The loss-of-function results demonstrated that HMOX1 silence reduced expression levels of ABCA6, ABCB1, ABCC1, and ABCG2 in these cells (Figure 8). Consistently, the gain-of-function results revealed that HMOX1 overexpression elevated mRNA levels of ABCA6, ABCB1, ABCC1, and ABCG2 in Huh7 and HepG2 cells (Figure 9).

### 3.6. The Prognostic Role of HMOX1 Expression in HCC Patients

**Figure 10:**
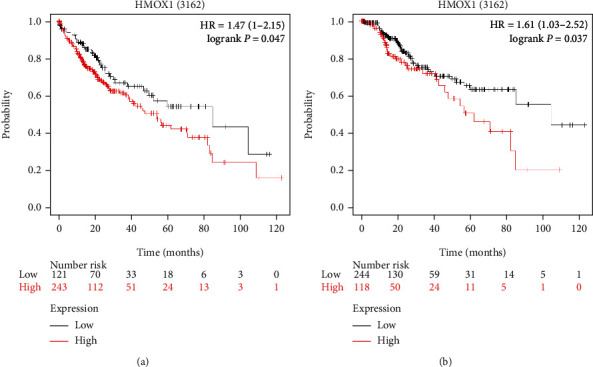
Actions of HMOX1 expression on OS and DSS of HCC patients. (a) Actions of HMOX1 expression on OS of HCC patients were examined by the KM-plotter. (b) Actions of HMOX1 expression on DSS of HCC patients were examined by the KM-plotter.

The effects of HMOX1 expression on the prognosis of HCC patients were determined by an online KM-plotter tool, and high expression of HMOX1 was associated with shorter OS and DSS in HCC patients (Figure 10).

## 4. Discussion

The acquired sorafenib resistance has significantly limited the therapeutic potential of sorafenib in advanced HCC, while mechanisms associated with acquired sorafenib resistance remain to be further clarified [10]. In this study, two RNA-sequencing datasets (GSE140202 and GSE143233) from GEO were downloaded for analysis. The DEGs between sorafenib-sensitive and the sorafenib-resistant group were extracted in these two datasets, and a total of 48 common DEGs between GSE140202 and GSE143233 were extracted. Functional enrichment analysis revealed that the common DEGs might be associated with “antigen processing and presentation,” “natural killer cell mediated cytotoxicity,” “cell adhesion molecules,” and so on. Ten hub genes were identified from the protein-protein interaction network based on common DEGs. Experimental results revealed the upregulation of HMOX1 in sorafenib-resistant HCC cells. HMOX1 silence promoted the sensitivity to sorafenib in sorafenib-resistant HCC cells; overexpression of HMOX1 attenuated the sensitivity. In addition, HMOX1 silence downregulated the mRNA expression of ABC transporters in sorafenib-resistant HCC cells, while HMOX1 overexpression upregulated mRNA expression of ABC transporter expression in HCC cells. Further analysis also revealed that high expression of HMOX1 was associated with shorter OS and DSS in HCC patients. Collectively, our results demonstrated that HMXO1 might be associated with sorafenib resistance in HCC.

Analysis of the microarray datasets has become a powerful tool for exploring novel genes that may be associated with cancer progression. In the GSE140202 dataset, TCNOS_00284048 and TCONS_00006019 were highly expressed in the sorafenib-resistant HCC cells compared with parental HCC cells. Knockdown of TCNOS_00284048 and TCONS_00006019 promoted sorafenib-sensitivity of Huh7-SR and HepG2-SR cells [22]. In GSE143233, Lin et al. demonstrated that METTL3 was underexpressed in human sorafenib-resistant HCC and revealed that RNA m^6^A methylation mediated sorafenib resistance via FOXO3-mediated autophagy [23]. Jiang et al. carried out integrated transcriptomic sequencing of GSE140202 and other datasets and identified 13 hub genes and seven promising therapeutic agents for HCC [18]. Li et al. also performed bioinformatics analysis for GSE140202 and found that GINS1 was highly expressed in sorafenib-resistant HCC cells [24]. In our results, we identified 50 common DEGs between GSE140202 and GSE143233, and these DEGs may be “antigen processing and presentation,” “natural killer cell mediated cytotoxicity,” “cell adhesion molecules,” and so on. Ten hub genes were detected from the PPI network. Among these hub genes, HMOX1 was highly expressed in sorafenib-resistant HCC cells as determined by qRT-PCR assay.

HMOX1 is well known for its enzymatic role in regulating cellular homeostasis under stress [25]. Increasing evidence has demonstrated the regulatory role of HMOX1 in cancer progression. For example, Yim et al. showed that HMOX1 is a prognostic marker for bladder cancer recurrence and progression [26]. Park et al. showed that the HMOX1/carbon monoxide axis inhibited transforming growth factor-*β*1-induced growth inhibition in HCC cells [27]. Inhibiting HMOX1 expression could retard HCC progression via distinct mechanisms [28–30]. However, the action of HMXO1 in HCC sorafenib resistance has not been reported yet. Gao et al. showed that inhibition of HMXO1 could sensitize clear-cell renal cell carcinoma to sorafenib [31]. Our study showed that HMOX1 was upregulated in the sorafenib-resistant HCC cells. HMOX1 could promote the resistance of HCC cells to sorafenib. Multidrug resistance is closely regulated by ABC transporters [32]. Studies have demonstrated that sorafenib can interact with ABC transporters such as ABCB1, ABCC1, ABCG2, and ABCC10 [33, 34]. In our study, sorafenib-resistant HCC cells exhibited high expression of ABC transporters, including ABCA6, ABCB1, ABCC1, and ABCG2 HCC cells. In addition, HMOX1 silence downregulated the mRNA expression of ABCA6, ABCB1, ABCC1, and ABCG2 in sorafenib-resistant HCC cells, while HMOX1 overexpression upregulated these transporters' expression in HCC cells. These results indicated that HMOX1-mediated sorafenib resistance might be associated with the modulation of the expression of ABC transporters.

## 5. Conclusions

In conclusion, the present study identified ten hub genes linked to the sorafenib resistance in HCC according to bioinformatics analysis. Further validation studies demonstrated that HMOX1 promoted the sorafenib resistance of HCC cells via modulating ABC transporter expression. However, the bioinformatic analysis was limited to two RNA-sequencing datasets, and future studies should explore more relevant datasets to identify more novel potential mediators in the regulating sorafenib sensitivity of HCC cells.

## Data Availability

All the data are available from the corresponding author.

## Supplementary Materials

Supplementary 1 Supplementary Table 1: siRNA sequences used in the study.

Supplementary 2 Supplementary Table 2: primer sequences for qRT-PCR analysis.

## References

[1] Marrero J. A. (2006). Hepatocellular carcinoma. *Current Opinion in Gastroenterology*.

[2] Wallace M. C., Preen D., Jeffrey G. P., Adams L. A. (2015). The evolving epidemiology of hepatocellular carcinoma: a global perspective. *Expert Review of Gastroenterology & Hepatology*.

[3] Fulgenzi C. A., D'alessio A., Talbot T. (2022). New frontiers in the medical therapy of hepatocellular carcinoma. *Chemotherapy*.

[4] García-Pras E., Fernández-Iglesias A., Gracia-Sancho J., Pérez-Del-Pulgar S. (2022). Cell death in hepatocellular carcinoma: pathogenesis and therapeutic opportunities. *Cancers*.

[5] Zhang H., Zhang W., Jiang L., Chen Y. (2022). Recent advances in systemic therapy for hepatocellular carcinoma. *Biomarker Research*.

[6] Yang J. D., Hainaut P., Gores G. J., Amadou A., Plymoth A., Roberts L. R. (2019). A global view of hepatocellular carcinoma: trends, risk, prevention and management. *Nature Reviews Gastroenterology & Hepatology*.

[7] Song T., Li L., Wu S. (2021). Peripheral blood genetic biomarkers for the early diagnosis of hepatocellular carcinoma. *Frontiers in Oncology*.

[8] Wei L., Lee D., Law C. T. (2019). Genome-wide CRISPR/Cas9 library screening identified PHGDH as a critical driver for sorafenib resistance in HCC. *Nature Communications*.

[9] Wu F. Q., Fang T., Yu L. X. (2016). ADRB2 signaling promotes HCC progression and sorafenib resistance by inhibiting autophagic degradation of HIF1*α*. *Journal of Hepatology*.

[10] Xia S., Pan Y., Liang Y., Xu J., Cai X. (2020). The microenvironmental and metabolic aspects of sorafenib resistance in hepatocellular carcinoma. *eBioMedicine*.

[11] Llovet J. M., Montal R., Sia D., Finn R. S. (2018). Molecular therapies and precision medicine for hepatocellular carcinoma. *Nature reviews Clinical oncology*.

[12] Tang W., Chen Z., Zhang W. (2020). The mechanisms of sorafenib resistance in hepatocellular carcinoma: theoretical basis and therapeutic aspects. *Signal Transduction and Targeted Therapy*.

[13] Weng H., Zeng L., Cao L. (2021). circFOXM1 contributes to sorafenib resistance of hepatocellular carcinoma cells by regulating MECP2 via miR-1324. *Molecular Therapy Nucleic acids*.

[14] Wu M. Y., Tang Y. P., Liu J. J., Liang R., Luo X. L. (2020). Global transcriptomic study of circRNAs expression profile in sorafenib resistant hepatocellular carcinoma cells. *Journal of Cancer*.

[15] Wu H., Wang T., Liu Y. (2020). Mitophagy promotes sorafenib resistance through hypoxia-inducible ATAD3A dependent Axis. *Journal of Experimental & Clinical Cancer Research*.

[16] Arechederra M., Bazai S. K., Abdouni A. (2021). ADAMTSL5 is an epigenetically activated gene underlying tumorigenesis and drug resistance in hepatocellular carcinoma. *Journal of Hepatology*.

[17] Liu J., Qiu W. C., Shen X. Y., Sun G. C., Department of Pharmacy, The Fifth People’s Hospital of Shanghai, Fudan University, Shanghai 200240, China (2019). Bioinformatics analysis revealed hub genes and pathways involved in sorafenib resistance in hepatocellular carcinoma. *Mathematical Biosciences and Engineering*.

[18] Jiang X., Zhang W., Li L., Xie S. (2021). Integrated transcriptomic analysis revealed hub genes and pathways involved in sorafenib resistance in hepatocellular carcinoma. *Pathology Oncology Research*.

[19] Mahi N. A., Najafabadi M. F., Pilarczyk M., Kouril M., Medvedovic M. (2019). GREIN: an interactive web platform for re-analyzing GEO RNA-seq data. *Scientific Reports*.

[20] Szklarczyk D., Gable A. L., Lyon D. (2019). STRING v11: protein-protein association networks with increased coverage, supporting functional discovery in genome-wide experimental datasets. *Nucleic Acids Research*.

[21] Lánczky A., Győrffy B. (2021). Web-based survival analysis tool tailored for medical research (KMplot): development and implementation. *Journal of Medical Internet Research*.

[22] Wu M., Shen X., Tang Y., Zhou C., Li H., Luo X. (2020). Identification and validation of potential key long noncoding RNAs in sorafenib-resistant hepatocellular carcinoma cells. *PeerJ*.

[23] Lin Z., Niu Y., Wan A. (2020). RNAm6A methylation regulates sorafenib resistance in liver cancer throughFOXO3‐mediated autophagy. *The EMBO Journal*.

[24] Li S., Wu L., Zhang H. (2021). GINS1 induced sorafenib resistance by promoting cancer stem properties in human hepatocellular cancer cells. *Frontiers in Cell and Developmental Biology*.

[25] Dulak J., Jozkowicz A. (2014). Novel faces of heme oxygenase-1: mechanisms and therapeutic potentials. *Antioxidants & Redox Signaling*.

[26] Yim M. S., Ha Y. S., Kim I. Y., Yun S. J., Choi Y. H., Kim W. J. (2011). HMOX1 is an important prognostic indicator of nonmuscle invasive bladder cancer recurrence and progression. *The Journal of Urology*.

[27] Park S. J., Lee S. K., Lim C. R. (2018). Heme oxygenase-1/carbon monoxide axis suppresses transforming growth factor-*β*1-induced growth inhibition by increasing ERK1/2-mediated phosphorylation of Smad3 at Thr-179 in human hepatocellular carcinoma cell lines. *Biochemical and Biophysical Research Communications*.

[28] Guoyin Z., Hao P., Min L., Wei G., Zhe C., Changquan L. (2017). Antihepatocarcinoma effect of Portulaca oleracea L. in mice by PI3K/Akt/mTOR and Nrf2/HO-1/NF-*κ*B pathway. *Evidence-Based Complementary and Alternative Medicine*.

[29] Zhang C., Li L., Hou S. (2021). Astragaloside IV inhibits hepatocellular carcinoma by continually suppressing the development of fibrosis and regulating pSmad3C/3L and Nrf2/HO-1 pathways. *Journal of Ethnopharmacology*.

[30] Zeng Y., Lian S., Li D. (2017). Anti-hepatocarcinoma effect of cordycepin against NDEA-induced hepatocellular carcinomas via the PI3K/Akt/mTOR and Nrf2/HO-1/NF-*κ*B pathway in mice. *Biomedecine & Pharmacotherapie*.

[31] Gao C., Peng F. H., Peng L. K. (2014). MiR-200c sensitizes clear-cell renal cell carcinoma cells to sorafenib and imatinib by targeting heme oxygenase-1. *Neoplasma*.

[32] Choi Y. H., Yu A. M. (2014). ABC transporters in multidrug resistance and pharmacokinetics, and strategies for drug development. *Current Pharmaceutical Design*.

[33] Brózik A., Hegedüs C., Erdei Z. (2011). Tyrosine kinase inhibitors as modulators of ATP binding cassette multidrug transporters: substrates, chemosensitizers or inducers of acquired multidrug resistance?. *Expert Opinion on Drug Metabolism & Toxicology*.

[34] van Erp N. P., Gelderblom H., Guchelaar H. J. (2009). Clinical pharmacokinetics of tyrosine kinase inhibitors. *Cancer Treatment Reviews*.

