# Supplementary material for: HMOX1 Attenuates the Sensitivity of Hepatocellular Carcinoma Cells to Sorafenib via Modulating the Expression of ABC Transporters
Source: Int J Genomics. 2022 Jun 27;2022:9451557. doi: 10.1155/2022/9451557 (PMC9253870; doi:10.1155/2022/9451557)
Supplement: Supplementary 1 — Supplementary Table 1: siRNA sequences used in the study. [file 9451557.f1.docx]

Supplementary Table 1. SiRNA sequences used in the study

| SiRNA name | Sequence (5'-3') |
| --- | --- |
| HMOX1 siRNA | GACUGCGUUCCUGCUCAAC |
| Scrambled siRNA | GGCAGUCCCCGUAUACUCU |
