# Supplementary material for: HMOX1 Attenuates the Sensitivity of Hepatocellular Carcinoma Cells to Sorafenib via Modulating the Expression of ABC Transporters
Source: Int J Genomics. 2022 Jun 27;2022:9451557. doi: 10.1155/2022/9451557 (PMC9253870; doi:10.1155/2022/9451557)
Supplement: Supplementary 2 — Supplementary Table 2: primer sequences for qRT-PCR analysis. [file 9451557.f2.docx]

Supplementary Table 2. Primers sequences for qRT-PCR analysis

| Primer name | Sequence (5'-3') |
| --- | --- |
| HMOX1 forward | TCAGGCAGAGGGTGATAGAAG |
| HMOX1 reverse | TTGGTGTCATGGGTCAGC |
| ABCA6 forward | AAACAGAAAAGCGTGTATCAGCA |
| ABCA6 reverse | AAACAGAAAAGCGTGTATCAGCA |
| ABCB1 forward | TTGGCTGATGTTTGTGGGAAG |
| ABCB1 reverse | CCAAAAATGAGTAGCACGCCT |
| ABCC1 forward | GTGAATCGTGGCATCGACATA |
| ABCC1 reverse | GCTTGGGACGGAAGGGAATC |
| ABCG2 forward | CAGGTGGAGGCAAATCTTCGT |
| ABCG2 reverse | ACCCTGTTAATCCGTTCGTTTT |
| GAPDH forward | TGTGGGCATCAATGGATTTGG |
| GAPDH reverse | ACACCATGTATTCCGGGTCAAT |
